# Clinical pattern and treatment outcome of cutaneous leishmaniasis patients in Somali region, eastern Ethiopia

**DOI:** 10.1002/ski2.416

**Published:** 2024-06-30

**Authors:** Abdilahi Ibrahim Muse, Muse Obsiye Ibrahim, Muse Ahmed Ibrahim

**Affiliations:** ^1^ Regional Data Management Center for Health Somali Region Health Bureau Jigjiga Ethiopia; ^2^ Department of Public Health Institute of Health Science Jigjiga University Jigjiga Ethiopia; ^3^ Department of Medicine Institute of Health Science Jigjiga University Jigjiga Ethiopia

## Abstract

**Background:**

Neglected tropical diseases, affecting over a billion people, include leishmaniasis, a protozoan parasite found in over 20 species. It is classified into three types: visceral, cutaneous, and muco‐cutaneous leishmaniasis, with cutaneous leishmaniasis being the most common. Dogs and rodents are the primary reservoirs of leishmaniasis, which is transmitted by infected female sandflies. Cutaneous leishmaniasis, found on exposed parts such as the arms, face and legs, presents with diverse clinical manifestations, including erythematous lesions, large, painless nodules, ulceration and an eventual volcanic form.

**Objective:**

To assess the clinical pattern and treatment outcome of cutaneous leishmaniasis patients in the Somali region of eastern Ethiopia.

**Methods:**

This descriptive study was conducted in the Somali region using neglected tropical disease surveillance data collected from 1 September 2023, to 8 February 2024. The data were cleaned and then exported to STATA version 17 for analysis.

**Results:**

From 1 September 2023, to 8 February 2024, 900 patients were diagnosed with cutaneous leishmaniasis. Of these, 105 (11.67%) patients had localised cutaneous leishmaniasis, 741 (82.33%) had disseminated cutaneous leishmaniasis, 20 (2.22%) had diffuse cutaneous leishmaniasis, 8 (0.89%) had mucocutaneous leishmaniasis, 12 (1.33%) had mucocutaneous and disseminated leishmaniasis and 14 (1.56%) had localised and mucocutaneous leishmaniasis. All of the cases were male; the most common signs and symptoms the patients showed were papule, nodule, ulcer and crust 791 (87.89%) and itching, fever and discharges 758 (84.22%), respectively. Most of the patients, 823 (93.34%), were given systemic pentavalent antimonials, and the cure rate was 886 (98.44%). Of the patients, 14 (100%) who were defaulted on had diffuse cutaneous leishmaniasis.

**Conclusions:**

All the participants had travelled to an area where sandflies were common. The majority of patients complained of itching, fever and discharges, with over two‐thirds having disseminated cutaneous leishmaniasis (DCL) and 0.89% having mucocutaneous leishmaniasis. The regional health bureau should set up a strong surveillance system and launch educational campaigns to raise awareness about cutaneous leishmaniasis, its transmission, symptoms and preventive measures. Furthermore, patients should be advised to strictly adhere to their treatment schedule and follow‐ups.

## Graphical abstract

Leishmaniasis, a protozoan parasite found in over 20 species, is one of the neglected tropical diseases that affects more than a billion people. A total of 900 people were diagnosed with cutaneous leishmaniasis. In total, 741 patients (82.33%) had disseminated cutaneous leishmaniasis. All the participants had been to an area where sandflies were prevalent. The majority of patients reported itching, fever, and discharges. The regional health bureau should establish a robust surveillance system and launch educational campaigns to increase awareness of cutaneous leishmaniasis, its transmission, symptoms and preventive measures. Additionally, patients should be advised to strictly follow their treatment schedule and follow‐ups.

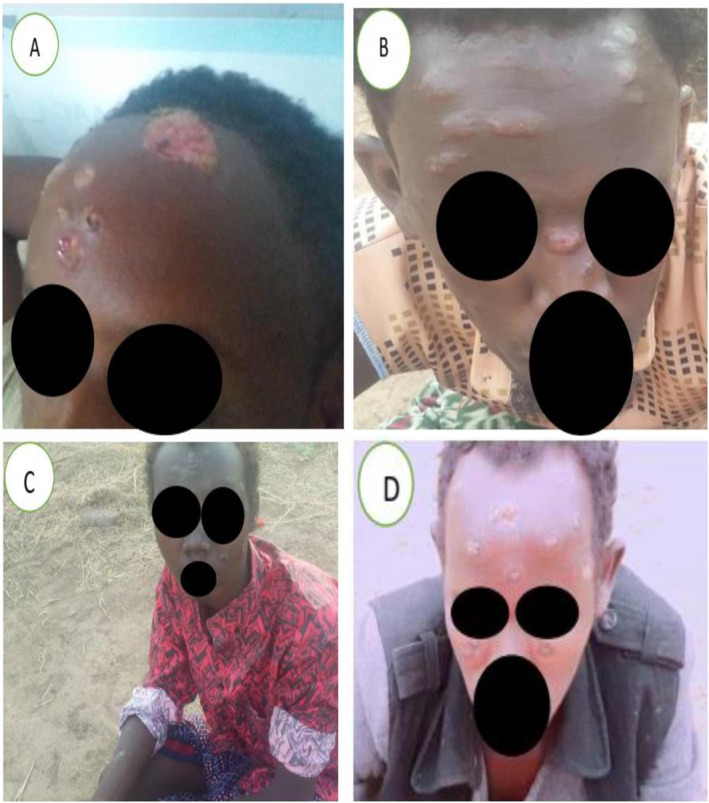

**What is already known about this study?**

- Cutaneous leishmaniasis is a significant public health concern that is caused by different protozoan species. Dogs and rodents are the primary reservoirs of leishmaniasis, which is transmitted by infected female sandflies.

**What does this study add?**

- The aim of this paper is to point out clinical patterns and treatment outcomes in cutaneous leishmaniasis patients in order to provide new perspectives on treatment strategies.

## 1 INTRODUCTION

Neglected tropical diseases (NTDs) include a wide‐ranging variety of circumstances caused by a range of pathogens, and it is estimated that NTDs affect over a billion people.^1^ Leishmaniasis is caused by a protozoan parasite found in over 20 different leishmania species, and classified into visceral leishmaniasis, cutaneous leishmaniasis and muco‐cutaneous leishmaniasis, with cutaneous leishmaniasis being the most common.^2^

Dogs and rodents are the primary reservoirs of leishmaniasis, which is transmitted by infected female sandflies.^3^ The clinical manifestations are extremely diverse, with unusual sites and atypical morphologies.^4^ The most commonly observed clinical type of lesion was papulo‐nodular, according to a study conducted in Sri Lanka.^5^ However, other studies found that nodulo‐ulcerative was the most common clinical type in different countries.^6^, ^7^, ^8^ While in Ethiopia, the commonest clinical presentation was reported to be plague.^9^

The lesion, initially erythematous, progresses to a large, painless nodule, which may ulcerate over 2 weeks to 6 months.^10^ Leishmaniasis is diagnosed by detecting leishmania parasites (or DNA) in tissue specimens using light microscopy of stained slides, molecular methods and specialised culture techniques.^11^, ^12^ Topical treatment is recommended for localised cutaneous leishmaniasis (LCL) patients with no mucosal advancement risk, while systemic therapy is recommended for mucocutaneous leishmaniasis (MCL), diffuse cutaneous leishmaniasis (DCL) and complicated LCL. Pentavalent antimonials and paromomycin are commonly used, while DCL is treated with a combination of them.^9^

Cutaneous leishmaniasis (CL) is a significant public health concern. Globally, the annual incidence of leishmaniasis is estimated to be 700 000 to 1 million cases, of which 20 000–30,000 deaths are attributed to it. Every year, approximately 66 941 patients are reported in the Americas.^13^ South America, the Mediterranean Basin, the Middle East and Central Asia account for about 95% of all cases of cutaneous leishmaniasis.^3^ Every state in Brazil has it, and between 72 800 and 119 600 people are impacted annually.^14^, ^15^, ^16^ Between 2014 and 2018, Ecuador reported 6937 leishmaniasis cases, with 97.5% being CL, with an average of 1395 cases per year.^17^ The majority of cases of cutaneous leishmaniasis occur in low‐income nations.^18^ For instance, in Pakistan, the spread of cutaneous leishmaniasis is causing increasing public health concern.^4^ Furthermore, in Sri Lanka, it has been reported that the free movement of infected people between conflict‐free areas contributes to the spread of the disease,^19^ while Iran and Syria are two of the six countries where cutaneous leishmaniasis is endemic and causes considerable morbidity and mortality.^20^

In Iran, the prevalence of infection has been reported to range from 1.8% to 37.9%,^21^ and it has the second highest annual incidence rate of 20 000 cases.^22^ In 2013, there were 1033 new cases reported in Syria, with 96.6% occurring among displaced Syrian refugees.^23^ Cutaneous leishmaniasis is widespread in North Africa, including Morocco, Algeria, Tunisia and Libya. Every year, the number of infected people increases.^24^ In Morocco, the average annual cutaneous leishmaniasis is 4165 cases.^25^ In addition, Algeria has the world's second‐highest incidence of cutaneous leishmaniasis, following Afghanistan.^26^ In Sudan, leishmania major is considered commonly responsible for cutaneous leishmaniasis, with 804 cases reported in 2016.^27^

According to Ethiopia, cutaneous leishmaniasis is a significant public health issue; approximately 30 million people are at risk, with 20 000 to 50 000 new cases reported annually.^28^ A study in the Amhara region revealed the highest human leishmaniasis prevalence (39.1%), while a survey in Tigray reported the lowest (2.3%).^29^ Cutaneous leishmaniasis was discovered to be complex and challenging to treat in north‐west Ethiopia.^30^ The country's severe ecological issues, including deforestation, overgrazing, soil erosion, desertification and environmental alteration susceptibility significantly impact the prevalence of vector‐borne infections.^31^

Despite the fact that various research and interventions have been done, leishmania remains one of the underreported tropical infectious diseases, and there are also few reports on cutaneous leishmaniasis in Ethiopia, particularly in the Somali region, where no study was conducted on this case and the population is primarily pastoralists who have no proper housing, with outside sleeping being common and limited access to hygienic and health facilities. This study aimed to assess the clinical pattern and treatment outcome of cutaneous leishmaniasis in the Somali region of eastern Ethiopia.

This study benefits individuals and communities affected by the disease by improving treatment, resulting in better patient outcomes, lower morbidity and a higher quality of life. It also provides healthcare professionals, such as physicians and nurses, with up‐to‐date evidence‐based knowledge about disease treatment and management. It provides insights for public health agencies, policymakers, researchers and scientists working on cutaneous leishmaniasis to advance knowledge in the field.

## 2 METHODS

### 2.1 Study setting and period

Ethiopia has 12 regions and 2 administrative cities, one of which is the Somali region. The region is located in the eastern part of Ethiopia and covers an area of over 375 000 km^2^. Its temperature ranges from 18 to 45 degrees Celsius, and its annual rainfall is from 386 to 660 mm. The Somali region has a population of 4.4 million people, according to the 2007 CSA. It is projected to be 6.8 million in 2015, with 86% living in rural areas and 14% in urban areas.^32^ Furthermore, there are 1596 health posts, 227 health centres and 18 hospitals in terms of health institutions. The study period was from 1 September 2023 to 8 February 2024.

### 2.2 Study design

This descriptive cross‐sectional study was conducted in the Somali region using NTD data.

### 2.3 Population

All populations living in the Somali region were the source population, while patients whose samples were detected to have parasites (confirmed and diagnosed) with cutaneous leishmaniasis were the study population.

### 2.4 Eligibility

This study included patients whose samples were detected to have parasites and treated in public hospitals in the Somali region and has completely filled line lists.

### 2.5 Data source

Secondary data (leishmania line list) from the Somali region's neglected tropical diseases and non‐communicable diseases directorate was used to study the clinical pattern and treatment outcome of cutaneous leishmaniasis in the Somali region. The principal investigator wrote a request letter to the neglected tropical diseases and non‐communicable diseases directorate, requesting the aforementioned data, and received it from the directorate. Furthermore, administrative reports and laboratory results from human samples collected during the outbreak period was used to summarise the outbreak. The leishmania line list included variables such as age, gender, residence, occupation, signs and symptoms, date of onset, date of admission, name of the health facility in which the case was treated, number of lesions, site of the lesion, treatment given and the outcome of the case.

### 2.6 Study variables

The variables studied were age, sex, travel history, place travelled to, patient characteristics, zones, districts, laboratory samples taken, type of sample taken, stain used, clinical signs, clinical symptoms, number of lesions, site of the lesions, type of cutaneous leishmaniasis, comorbidity, type of comorbidity, treatment initiation and the treatment option.

### 2.7 Data quality checks

Since the data was in a line list, accuracy, consistency, completeness, and validity was all checked.

### 2.8 Operational definitions

**Cases**: patients diagnosed with cutaneous leishmaniasis.

**Cured**: Patients who, after 3 months of treatment, have shown a clinical response (re‐epithelialisation and flattening of the lesion) and/or parasitological confirmation of recovery.^9^

**Defaulter**: is a person who, although capable of doing so, declines, postpones or ends a course of treatment.

### 2.9 Data processing and analysis

The collected data were checked for completeness and consistency. The data were cleaned and exported to STATA version 17 software for analysis. Descriptive statistics was computed using the measures of the central tendency, frequency and percentages, and then the clinical pattern of the disease was summarised.

## 3 RESULTS

### 3.1 Socio‐demographic characteristics of cutaneous leishmaniasis patients in the Somali region, eastern Ethiopia

The study included nine hundred (900) participants, all of whom were male and had travelled to an area where sandflies are highly prevalent. Age ranged from 15 to 63 years, with a mean of 25.6 (±SD 6.86). The vast majority of the participants, 899 (99.89%), were police officers, with only one (0.11%) being a civilian. The majority of the participants were from Faafan Zone comprising 331 participants (36.78%), followed by Sitti Zone comprising 224 participants (24.89%). Furthermore, based on the district report, the majority of the cases were from (36.78%) Jigjiga city comprising 331 participants and 161 (17.89%) participants were from Gota‐Biki (Table 1).

**TABLE 1:** Socio‐demographic characteristics of cutaneous leishmaniasis patients in the Somali region, eastern Ethiopia.

Variable	Category	Frequency	Percent
Age in years	≤18	53	5.89
	19–28	627	69.67
	29–38	166	18.4
	≥ 39	54	6.00
Age in years	Mean, ±SD	25.6 (±SD 6.86).	
Sex	Male	900	100
Travel history	Yes	900	100
Place travelled to	East Sitti zone	900	100
Patient characteristic	Police	899	99.89
	Civilian	1	0.11
Zone	Afder	39	4.33
	Dawa	150	16.67
	Dollo	8	0.89
	Liban	52	5.78
	Sitti	224	24.89
	Faafan	331	36.78
	Shabelle	96	10.67
Districts	Dollo‐ado	32	3.56
	Filtu	20	2.22
	God‐god	39	4.33
	Gota‐biki	161	17.89
	Jigjiga city	331	36.78
	Shinile	63	7.00
	Gode	96	10.67
	Moyale	150	16.67
	Warder	8	0.89

### 3.2 Laboratory and clinical features of cutaneous leishmaniasis patients in the Somali region of eastern Ethiopia

Based on the laboratory and the samples, samples were taken from all of the patients, and 900 (100%) of the participants tested positive. Of the samples taken from the participants, more than two‐thirds were from Fine Needle Aspiration Cytology (FNAC). All of the samples collected were stained with gram stain. Regarding the clinical signs and symptoms of the patients, in more than two‐thirds of the cases, 791 (87.89%) had a papule, nodule, ulcer or crust, and 758 (84.22%) had itching, fever and discharges, respectively. Furthermore, based on the location of the lesion, 803 (89.22%) of the participants had lesions on their faces, necks or limbs. Lesions in cutaneous leishmaniasis patients ranged from 2 to 73, with an average number of 16.12 (± SD 8.43). The majority of them, 859 (95.44%), had more than seven lesions, whereas 803 (89.22%) participants had lesions on the face, neck and limbs (Table 2).

**TABLE 2:** Laboratory and clinical features of cutaneous leishmaniasis patients in the Somali region.

Variable	Category	Frequency	Percent
Laboratory samples taken	Yes	900	100
Type of sample taken	FNAC	651	72.33
	Biopsy	20	2.22
	Wound tissue scrap	80	8.89
	Skin snip	149	16.56
Stain used	Gram stain	900	100
Clinical signs	Nodule	16	1.78
	Ulcer	1	0.11
	Papule and nodule	13	1.44
	Papule, nodule and ulcer	25	2.78
	Papule, nodule, ulcer and crust	791	87.89
	Ulcer and crust	38	4.22
	Nodule and ulcer	12	1.33
	Nodule, ulcer and crust	4	0.44
Clinical symptoms	No symptoms	5	0.56
	Itching	3	0.33
	Fever	1	0.11
	Discharge	3	0.33
	Itching and fever	10	1.11
	Itching, fever and discharges	758	84.22
	Itching and discharge	109	12.11
	Fever and discharge	11	1.22
Number of lesions	2–4 lesions	18	2.00
	5–6 lesions	23	2.56
	≥7 lesions	859	95.44
Site of the lesions	Face	2	0.22
	Lower limbs	2	0.22
	Face, neck and limbs	803	89.22
	Face and neck	26	2.89
	Face and limbs	55	6.11
	Face and lower limbs	5	0.56
	Limbs	7	0.78

**FIGURE 1:**
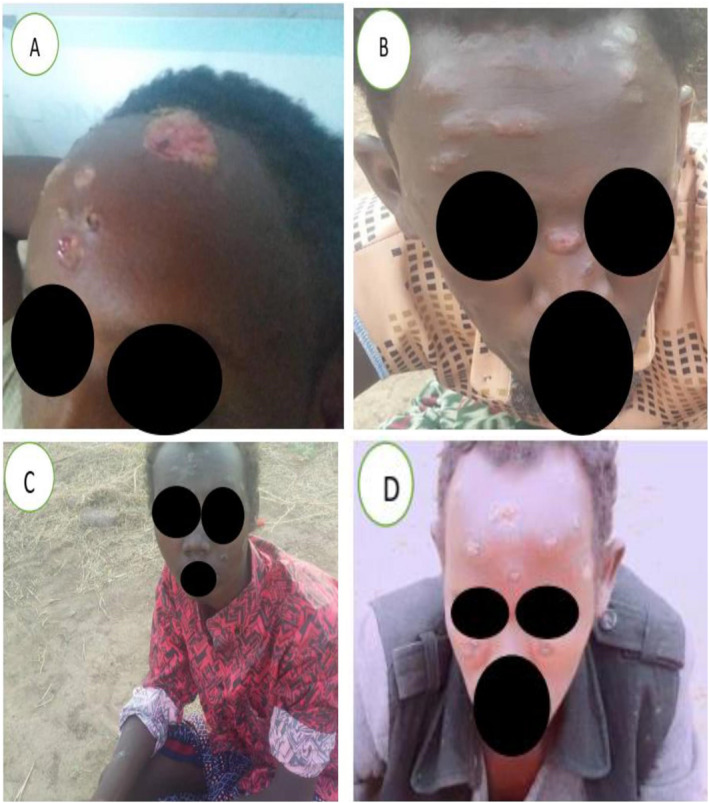
(a) An open, painful ulcer with uneven edges; the surrounding area is inflamed, with redness and swelling. (b) An inflammatory ulcer on the forehead that has an open, raw core and an uneven elevated border. (c) The lesion is large, open and infectious, with visible pus and debris, indicating that the disease is severe and has progressed. The adjacent skin is also discoloured and tough, indicating a chronic inflammatory response. (d) The man's face had elevated, red and inflammatory lesions. They appear to have crusty ulcers. The lesions range in size and shape, and they are spread across the man's face, including his cheeks and chin.

A. an open, painful ulcer with uneven edges; the surrounding area is inflamed, with redness and swelling; B. an inflammatory ulcer on the forehead that has an open, raw core and an uneven, elevated border; C. The lesion is large, open and infectious, with visible pus and debris, indicating that the disease is severe and has progressed. The adjacent skin is also discoloured and tough, indicating a chronic inflammatory response; D. The man's face had elevated, red and inflammatory lesions. They appear to have crusty ulcers. The lesions range in size and shape, and they were spread across the man's face, including his cheeks and chin (Figure 1).

### 3.3 Type of cutaneous leishmaniasis, its comorbidity, their treatment options and the treatment outcome of cutaneous leishmaniasis patients in the Somali region

Based on the cutaneous leishmaniasis, its comorbidity and treatment options of cutaneous leishmaniasis patients, more than two‐thirds of them, 741 (82.33%), had diffuse cutaneous leishmaniasis, and only 9 (1%) of them had comorbidity. Separately, one‐third of those with the comorbidity, 3 (33.3%), had jaundice and other comorbidities each. All of the patients were given medication, and according to the route of administration, more than half of the patients 498 (55.33%) received their medication intramuscularly. Furthermore, most of the patients, 886 (98.44%), were cured, while 14 (1.56%) of them defaulted (Table 3).

**TABLE 3:** Type of cutaneous leishmaniasis, its comorbidity and the treatment options of cutaneous leishmaniasis patients in the Somali region.

Variable	Category	Frequency	Percent
Type of cutaneous leishmaniasis	LCL	105	11.67
	DCL	741	82.33
	DFCL	20	2.22
	MCL	8	0.89
	DCL & MCL	12	1.33
	LCL & MCL	14	1.56
Comorbidity	Yes	9	1
	No	891	99
Type of comorbidity	DM	1	11.11
	HTN	2	22.22
	Jaundice	3	33.33
	Others	3	33.33
Treatment initiation	Yes	900	100
Treatment option	SSG through IV	343	38.11
	SSG through IM	498	55.33
	GV through topical route	59	6.56

### 3.4 Treatment outcome of cutaneous leishmaniasis by socio‐demographic, clinical features, cutaneous leishmania type, its comorbidity, and treatment options of cutaneous leishmaniasis patients in the Somali region

According to the socio‐demographic characteristics and treatment outcomes of cutaneous leishmania patients, the highest default rate occurred in the 19–28 years age group with 11 patients (78.53%). Furthermore, patients with clinical signs of papule, nodule, ulcer or crust had the highest default rate of 10 patients (71.43%). According to the type of cutaneous leishmaniasis, 14 (100%) of those who defaulted had disseminated cutaneous leishmaniasis and received medication via intravenous route (Table 4).

**TABLE 4:** Treatment outcome of cutaneous leishmaniasis by socio‐demographic, clinical features, cutaneous leishmania type, its comorbidity and treatment options of cutaneous leishmaniasis patients in the Somali region.

Variables	Category	Treatment outcome	
		Cured, *N* (%)	Defaulted, *N* (%)
Age in years	≤18	52 (5.87)	1 (7.14)
	19–28	616 (69.53)	11 (78.53)
	29–38	164 (18.51)	2 (14.29)
	≥ 39	54 (6.09)	0 (0.00)
Clinical signs	Nodule	16 (1.81)	0 (0.00)
	Ulcer	0 (0.00)	1 (7.14)
	Papule and nodule	13 (1.47)	0 (0.00)
	Papule, nodule and ulcer	25 (2.82)	0 (0.00)
	Papule, nodule, ulcer and crust	781 (88.15)	10 (71.43)
	Ulcer and crust	35 (3.95)	3 (21.43)
	Nodule and ulcer	12 (1.35)	0 (0.00)
	Nodule, ulcer and crust	4 (0.45)	0 (0.00)
Type of cutaneous leishmaniasis	LCL	105 (11.85)	0 (0.00)
	DCL	727 (82.05)	14 (100)
	DFCL	20 (2.26)	0 (0.00)
	MCL	8 (0.98)	0 (0.00)
	DCL and MCL	12 (1.35)	0 (0.00)
	LCL and MCL	14 (1.58)	0 (0.00)
Comorbidity	Yes	9 (1.02)	0 (0.00)
	No	877 (98.98)	14 (100)
Treatment option	SSG through IV	329 (37.13)	14 (100)
	SSG through IM	498 (56.21)	0 (0.00)
	GV through topical route	59 (6.66)	0 (0.00)

## 4 DISCUSSION

The Somali region in eastern Ethiopia is experiencing its first outbreak of cutaneous leishmaniasis. The study's participants were almost all police officers 899 (99.89%) who travelled to the eastern Sitti zone, where sandflies are active and abundant. All participants provided samples, which were confirmed positive. Most patients had more than seven lesions on their faces, necks and limbs.

In this study, it has been found that cutaneous leishmania distributions were high among participants aged 19–28 years, which is in line with a study conducted in Brazil.^14^ In this study, the majority of patients with co‐morbidity (77.8%) were in this age range. This emphasises the notion that patients with comorbid disorders may bear the burden of immune system deficiency. The immune response to leishmania parasites plays a crucial role in determining the outcome of the infection. Moreover, although more than eighty percent of the study participants had diffuse cutaneous leishmaniasis, mucocutaneous involvement was also noted in this study, which is in line with another study that was conducted in Colombia.^33^ MCL is a more severe and destructive form of leishmaniasis that affects the mucous membranes.

Based on the site of the lesion, the majority of the patients had it on the face, neck and limbs, but another study carried out in Saudi Arabia reported that 90% of the patients had the lesion on their faces.^34^ The reason is that in this study, all of the patients were sleeping outside because of their working conditions and environment. Moreover, the face, neck and limbs were the exposed parts of their bodies, which might make it easy for the sandflies to bite on their face, neck and limbs. Furthermore, according to the clinical signs of the patients, a study in Iran reported that the majority of the patients had papule, while this study found that more than two‐thirds of the patients had papule, nodule, ulcer and crust.^10^ This might be because patients in Iran sought treatment as early as possible, earlier than those studied in this study because they did not have time or access to seek health care due to their working conditions.

According to the treatment, all of the patients were initiated with treatment and were given sodium stibogluconate (SSG) through intravenous (IV) or intramuscular (IM) and Gentian Violet; only 59 patients (6.59%) received Gentian Violet, and the remaining received sodium stibogluconate. Therefore, it has been found that the cure rate was very high at 98.44%. This finding is in line with some other studies that were carried out in southern and eastern Ethiopia.^35^ The reason for this inclusion is that the patients received similar courses of treatment, which could make them have a similar treatment outcome.

## 5 CONCLUSION AND RECOMMENDATIONS

According to the study, almost all the participants were police officers who had travelled to an area where sandflies were common. After exhibiting some signs and symptoms, samples were taken from each participant, and leishmaniasis was confirmed in all of them. The participants' mean age was 25.6 (±SD 6.86) years. The majority of the patients complained of itching, fever and discharges in addition to having papules, nodules, ulcers and crusts. Additionally, based on evaluation, the majority of the patients had lesions totalling more than seven. More than two‐thirds of the participants had disseminated cutaneous leishmaniasis (DCL), whereas only eight patients (0.89%) had mucocutaneous leishmaniasis. Based on the patients' health status, only 9 patients (1%) had a comorbid disease, which may have caused or contributed to a higher cure rate.

Given that cutaneous leishmaniasis is the first outbreak in the Somali region, it is recommended that the regional health bureau and its stakeholders establish a robust surveillance system to monitor the incidence and prevalence of cutaneous leishmaniasis cases in the region. Create educational campaigns to raise awareness among the general public, healthcare professionals and at‐risk populations, as well as provide information on the mode of transmission, symptoms, preventive measures and treatment options.

Emphasise the importance of early detection and treatment in order to avoid complications and transmission. Implement vector control measures to reduce the number of sandflies, the primary vectors of leishmaniasis. In addition, patients should be advised to strictly adhere to their treatment schedule and follow‐ups. Research is needed to better understand the local epidemiology, vectors and reservoirs of cutaneous leishmaniasis in the region.

### 5.1 Limitations of the study

The limitations of the study are the nature of the study design and the secondary data analysed. Another constraint of this study is the homogeneity of the participants, which might affect the generalisability of the findings.

## CONFLICT OF INTEREST STATEMENT

None to declare.

## AUTHOR CONTRIBUTIONS

**Abdilahi Ibrahim Muse**: Conceptualisation (equal); Data curation (lead); Formal analysis (equal); Investigation (lead); Methodology (lead); Project administration (equal); Resources (lead); Software (lead); Supervision (lead); Validation (lead); Writing – original draft (lead); Writing – review & editing (lead). **Muse Obsiye Ibrahim**: Conceptualisation (equal); Resources (equal); Software (equal); Supervision (equal); Validation (equal); Visualisation (equal); Writing – original draft (equal); Writing – review & editing (equal). **Musse Ahmed Ibrahim**: Conceptualisation (equal); Data curation (equal); Formal analysis (equal); Investigation (equal); Methodology (equal); Project administration (equal).

## ETHICS STATEMENT

The Somali region NTD directorate provided data for this study. The Somali Region Research and Laboratory Services Committee granted ethical approval (ref. SRHB/NTD/01/2024) for the use of the patient's data.

## PATIENT CONSENT

Written patient consent for publication was obtained.

## Data Availability

The data underlying this article will be shared on reasonable request to the corresponding author.
